# Adoption and sustained use of cleaner cooking fuels in rural India: a case control study protocol to understand household, network, and organizational drivers

**DOI:** 10.1186/s13690-017-0244-2

**Published:** 2017-12-14

**Authors:** Praveen Kumar, Amar Dhand, Rachel G. Tabak, Ross C. Brownson, Gautam N. Yadama

**Affiliations:** 1 Boston College School of Social Work, Boston College, 125 McGuinn Hall, 140 Commonwealth Avenue, Chestnut Hill, MA 02467 USA; 2 Department of Neurology, Harvard Medical School/Brigham and Women’s Hospital, 75 Francis Street, Boston, MA 02115 USA; 3 Network Science Institute, Northeastern University, 177 Huntington Street, Boston, MA 02115 USA; 4 Brown School, Washington University in St. Louis, Campus Box 1196, One Brookings Drive, St. Louis, MO 63130 USA; 5 Boston College School of Social Work, McGuinn Hall 132, 140 Commonwealth Avenue, Chestnut Hill, MA 02467 USA

**Keywords:** Implementation science, LPG, Cleaner cooking systems, Re-aim, Adoption, Sustained use, Implementation strategies, Gender-based networks, Stove Use Monitoring Systems

## Abstract

**Background:**

Implementing efficient stoves and clean fuels in low and middle-income countries are critical for improving health of poor women and children and improve the environment. Cleaner biomass stoves, however, perform poorly against the World Health Organization’s indoor air quality guidelines. This has shifted the focus to systematic dissemination and implementation of cleaner cooking systems such as liquefied petroleum gas (LPG) among poor communities. Even when there is some uptake of LPG by poor communities, its sustained use has been low. Concurrent use of LPG with traditional biomass cookstoves compromises reductions in household air pollution and limits health and environmental dividends. Therefore understanding key drivers of adoption and sustained implementation of clean fuels among the poor is critical. There is a significant gap, however, in the research to understand determinants and sustained exclusive use of clean fuels in rural poor communities.

**Methods/design:**

Using a case control study design, this study will explore the impact of affordability, accessibility, and awareness on adoption and sustained use of LPG among rural poor communities of India. The study uses a multistage random sampling to collect primary data from 510 households. Case group or LPG adopters constitute 255 households while control group or non-LPG adopters constitute the remaining 255 households. The study will deploy sophisticated stove use monitoring sensors in each of the stoves in 100 case group households to monitor stove use and stacking behavior (using clean and traditional systems of cooking) of participants for 12 months. Moreover, this will be the first study to explore the impact of personal social networks striated by gender on LPG adoption. This study is guided by the RE-AIM (Reach, Effectiveness, Adoption, Implementation, and Maintenance) implementation science evaluation framework.

**Discussion:**

Lessons from this study will feed into a larger discussion on developing a pro-poor strategy to foster uptake and sustained use of cleaner cooking systems such as LPG. Understanding the determinants of adoption and sustained use of cleaner cooking systems through the RE-AIM framework will expand our insights on implementation of cleaner cooking systems among poor communities and will advance implementation science in the clean cooking sector. A thorough study of such implementation strategies is crucial to realize multiple UN Sustainable Development Goals on global health, climate change, and energy security.

## Background

### Challenges with cleaner biomass stoves

The UN commitment to achieving 17 global goals by 2030 includes “access to affordable, reliable, sustainable, and modern energy for all” (Global Goal 7). This goal recognizes the harmful impacts of household air pollution (HAP) on almost 41% of the global population, predominantly poor, who continue to rely on solid fuels such as biomass, crop residues, and dung, for heating and cooking [1]. Owing to poor combustion efficiency, these solid fuels release aerosol emissions and particulate matters. They are a major source of HAP. These emissions have a detrimental impact on health, climate, and environment. Particularly, poor women and children are at a high risk of exposure to biomass smoke and adverse health outcomes causing acute and chronic respiratory infection [2]. Approximately 4.3 million annual premature deaths are attributed to HAP exposure [3]. Nearly 50% deaths from acute lower respiratory infection among children below 5 years in underdeveloped countries are attributed to exposure to HAP [3]. Continuous exposure to these emissions also leads to pregnancy complications and stunted growth of children [3]. In 2013, slightly more than 900,000 deaths have been attributed to HAP in India [4, 5].

Adoption and sustained use of cleaner cooking technologies such as cleaner biomass stoves or cleaner fuels (Liquefied Petroleum Gas [LPG]) are recommended as solutions to address the challenge of HAP. Dissemination and implementation (D&I) in the clean cooking sector has most recently focused on cleaner biomass stoves [6–9] . Strategies to disseminate and implement these cleaner biomass stoves among poor communities are motivated by four realizations: 1) a supra-linear nature of the HAP exposure-response curve suggests that expected health benefits of clean cooking can be attained only at very low levels of exposure [10, 11]. This means that health benefits can be derived only when biomass stoves reduce emissions from combustion to extremely low levels. However, most of the cleaner biomass stoves in use or being developed perform poorly against the World Health Organization’s (WHO) recommended indoor air quality guidelines (IAQG). Exposure levels with these cleaner biomass stoves during routine use in households are far higher than the recommended IAQG. Therefore health related benefits are not as forthcoming despite switching to cleaner biomass stoves; 2) communities have to continue to perpetually depend on biomass as a cooking fuel to use cleaner biomass stoves. These stoves do not offer a sustainable solution in terms of degradation of forests due to continued biomass harvest for use in these stoves for cooking and heating [8]; 3) drudgery of collecting biomass and associated physical injuries continue to pose health and economic challenges. Poor community members (especially women) have to travel long distances to collect biomass. Injuries from carrying heavy logs of wood and biomass are common among women in rural communities; 4) most of the cleaner biomass stoves developed in laboratories have poor performance in terms of robustness and mechanical wear and tear in real world conditions. Thus, despite some promise offered by these cleaner biomass stoves, health and environmental benefits are substantially compromised. It is increasingly recognized that while efforts are required to develop standards and technology for cleaner biomass burning, more emphasis is needed to deploy cleaner cooking systems such as LPG.

### Previous research on adoption and sustained use of LPG: Key limitations

Available literature on LPG use by poor communities can be broadly analyzed along supply and demand side of LPG.

#### Supply of LPG for poor households

Studies on LPG from the supply point of view have mostly focused on subsidies, pro-poor financing techniques, and low cost supply chain to increase affordability of LPG adoption and use for poor households. LPG, a clean and modern household fuel is a petroleum product and its price is mostly governed by fluctuations in international markets [12]. Nevertheless, it continues to outpace increase in income of poor communities [6, 12]. Blanket fuel subsidies provided by governments such as India reduce direct costs of acquiring LPG by households. However, such subsidies have not been successful as a policy instrument to increasing supply in poor communities. Poor households account for only a small part of total LPG fuel use as compared to their wealthy counterparts [8, 9, 13]. Supply of clean fuels such as LPG to billions of poor communities requires high-level policy initiatives. It involves careful trade negotiations and a mechanism of differential subsidies [8, 9]. Streamlining subsidies of LPG to benefit poor communities is a gradual process [14] involving multiple stakeholders (petroleum companies, petroleum rich nations, and national government subsidies) with conflicting interests [15]. It may also require an overhauling of energy policy at a higher level [12]. Large-scale studies of the supply of LPG are significant to address the challenge. Streamlining of subsidies, low cost supply chain mechanisms, and pro-poor financing techniques have the potential to make LPG more affordable for poor households [15]. However, even if the government attains an enabling supply-side climate, uptake and sustained use of LPG remains a distant goal if there is a limited demand from poor communities. Smith and Sagar [8] and Slaski and Thurber [16] argue that commensurate demand may expedite both rationalization of subsidies and revamp of energy policy for poor communities. A stronger evidence base on how to stimulate LPG demand among poor is needed [8, 12, 13, 17, 18].

#### Demand of LPG by poor households

There are limited and scattered studies focusing on analyzing the demand of LPG by poor households and most of the studies have combined different cleaner cooking technologies (including LPG). There has been growing attention to exploring causality between affordability of households and adoption of LPG. For instance, the majority of the empirical literature on adoption of cleaner cooking systems (including LPG) have three variables in common in their analyses: 1) household size; 2) income; and 3) fuelwood price [19]. The relationship between income and adoption is moderated by social class, gender, acquisition barriers, and ethnicity. Female-headed households with higher incomes are more likely to adopt cleaner cooking technologies. In patriarchal societies even if the households have higher income, they are less likely to adopt LPG [20]. Households belonging to marginalized groups, lower castes,^1^lower social class, or indigenous groups are less likely to adopt LPG [19].

Existing literature on adoption and sustained use of LPG by poor communities has three limitations. They are: 1) LPG is not a primary fuel in a majority of poor communities. Stacking clean fuels with traditional cooking technologies is common, which limits expected health and environmental dividends [21]. Limited systematic studies are available to explore this challenge of stacking and many do not account for stacking in a methodical way; 2) successful cases of poor households who have sustainably used LPG are needed. Lessons from such cases can then be adapted and tailored for other poor communities; 3) there is considerable attention to understanding the impact of affordability on LPG use. Increase in affordability to purchase LPG may drive uptake and sustained use. Lewis, Bhojvaid [22] argue that even if the acquisition barriers (like upfront cost) are waived to make cleaner technologies more affordable, sustained and exclusive use is low. Increase in affordability is a significant, however, an inadequate driver. Limited accessibility and awareness restrains communities from transition to and sustained use of LPG [16, 23, 24]. There is limited evidence exploring combined issues of affordability, accessibility, and awareness (3As) and their relation to adoption and sustained use of LPG by poor communities [25, 26].

## Methods

### Study aim

This paper is part of our larger study aimed at exploring the determinants of adoption and sustained use of LPG in resource poor communities of rural India. The study is guided by the RE-AIM (Reach, Effectiveness, Adoption, Implementation, and Maintenance) implementation sciences framework. This protocol paper highlights the application of the RE-AIM framework in our study of LPG implementation in poor households. The overarching goal of our study is to derive new insights on the reach of LPG among the poor in rural India, factors that influence adoption (initial uptake), sustained use, and maintenance of LPG in below poverty line (BPL) households in rural India. Our specific aims are:

1. To understand how below poverty LPG adopters vary from other BPL households on factors of affordability, accessibility, and awareness of LPG.
2. To determine how affordability, accessibility, and awareness affect sustained and exclusive use of LPG in adopter households.
3. To evaluate the relative influence of gender networks on LPG adoption and sustained use in BPL households.

### Study design

**Fig. 1:**
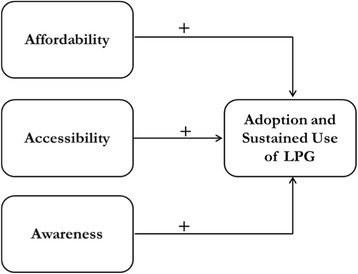
Impact of 3As on adoption and sustained use of LPG. This figure is a visual representation of the conceptual model arguing the concurrent role of affordability, accessibility, and awareness in fostering adoption and sustained use of LPG in poor communities. This study will test the validity of this model

Our study will employ a quantitative case-control study approach [27]. Adoption and sustained use of technology (such as LPG) by poor communities has a long latency period, and is impacted by multiple parameters from social, economic, and technological domains [28]. Case-control studies are suited for such phenomena, which have a long latency period [27]. These studies are relatively inexpensive to implement, and allow for concurrent analysis of multiple determinants [27]. Our study will test the conceptual model shown in Fig. 1.

Different stove adoption related studies have defined adoption and use in ways specific to their studies. It is imperative to define the five concepts illustrated in Fig. 1. Definitions of these concepts as they pertain to this study are:

1. *Awareness* refers to the degree of knowledge and perception about LPG adoption and use [19]. Several studies recognize the significance of awareness in motivating households for a fuel switch [19, 22]. Scattered evidence suggests that low information and scattered rumors on LPG safety issues may act as a deterrent to uptake and use of LPG by these rural households [23]. Measures of affordability, accessibility, and awareness are predictors of outcome variables of adoption (aim 1) and sustained use (aim 2) in this study. To further explore impact of awareness, we will also conduct social network analysis (aim 3) to assess how knowledge and awareness flows through social networks and consequently impacts the decision to adopt LPG among households.
2. *Accessibility* of LPG indicates factors impacting a household’s ability to procure LPG cylinders and stoves when needed. Factors affecting accessibility include (but are not limited to) distance to rural LPG distribution center, delivery mechanism of LPG cylinders, and road connectivity from villages to local distribution centers [23].
3. *Affordability* refers to the maximum possible capacity of households to pay for the minimum level of services [23]. Affordability is impacted by household factors such as income and LPG demand, and also by government policies on LPG subsidies [23].
4. *Adoption* refers to the initial uptake of LPG [29, 30]. Adoption of LPG is independent of the behavioral phenomena of sustained use of LPG or stacking LPG with traditional stoves. Adoption of LPG is a dichotomous outcome variable (LPG adopter households/LPG non adopter households) in this study (aim 1).
5. *Sustained use* shows the degree to which LPG is used and has been integrated in daily behavior of users [29, 30]. Sustained users who exclusively use LPG make a complete switch to LPG with no intention of reverting to traditional stoves or traditional fuels [29]. Stackers are those households who combine use of LPG with traditional stoves. We study the degree of use of each of these stoves for a select number of households (from group: LPG adopters) by using the Stove Use Monitor Systems (SUMS) technology. SUMS are based on commercially available temperature loggers, which record the variation in the stove temperature over a defined period of time to provide accurate insights into stove usage patterns, duration of use, and number of meals cooked. There are numerous models of SUMS available. For this study we are using iSUMS model DS 1922 L, which can record data for as long as 4–6 weeks post installation on the stoves [31]. Sustained use of LPG is a continuous outcome variable (aim 2). We measure this variable with the help of SUMS technologies, which will record the number of hours of use of LPG stoves and of traditional stoves. For analysis, we can also dichotomize sustained use into two categories: exclusive LPG users, and stackers. Households which use LPG at least 80% of the total cooking duration during our monitoring period (recorded with SUMS technology) will be characterized as exclusive LPG users. Such a metric will allow us to compare the results of this study with other LPG adoption studies now underway in different parts of the world [32];

### RE-AIM evaluation framework

RE-AIM is a systematic framework, which expands the assessment of a social or a public health intervention, evaluates its potential for translating research into practice and policy, and bridges the research-practice gap [33, 34]. The different aims of our study find a larger meaning and value when framed within the RE-AIM framework. RE-AIM stands for **R**each, **E**ffectiveness, **A**doption, **I**mplementation, and **M**aintenance. The five dimensions of the RE-AIM framework provide a way to synthesize the findings from across our three aims of the study: 1) **R**each is a measure of participation [34]. It refers to the proportion of the target population that has participated in the intervention [34]. Reach is concerned with the characteristics of the participants and whether they truly represent the target population [33, 34]. In understanding the rural poor LPG users now being reached, we stand to gain insights on how the program may improve its reach to larger proportion of rural poor households. We collect these demographic characteristics in aim 1 of the study; 2) **E**ffectiveness refers to the success rate of the health intervention, if implemented [33, 34]. The evidence based technology in our study is LPG. The effectiveness of LPG has been established by the WHO. LPG meets all the required IAQG [8]; 3) **A**doption refers to the absolute number or proportion of the target population who take up an evidence based health intervention [34]. Adoption is usually assessed by direct observation or structured interviews [34]. We examine adoption of LPG in aim 1 of the study. We examine affordability, accessibility, and awareness (3As) in aim 1 and relative influence of gender networks on LPG adoption in aim 3, to examine the predictors impacting adoption of LPG [9, 23, 35]; 4) **I**mplementation refers to fidelity in LPG use, and adherence of distribution programs in LPG delivery as intended [33, 34, 36]. Through structured interviews, we examine the 3As in aim 1 to assess if the LPG distributors reliably provide LPG permits and cooking fuel to meet the needs of the participants, as per demand and within policy and program guidelines; 5) **M**aintenance measures the extent to which the intervention has been integrated into the routine practices of the participants [34]. It is accompanied by a change in the practice patterns of the participants to sustainably integrate the health intervention in their routine practices without any intention of abandonment [34]. In this study, we assess maintenance in aim 2 by deploying SUMS technologies over 12 months to examine the extent of sustained use of LPG.

**Fig. 2:**
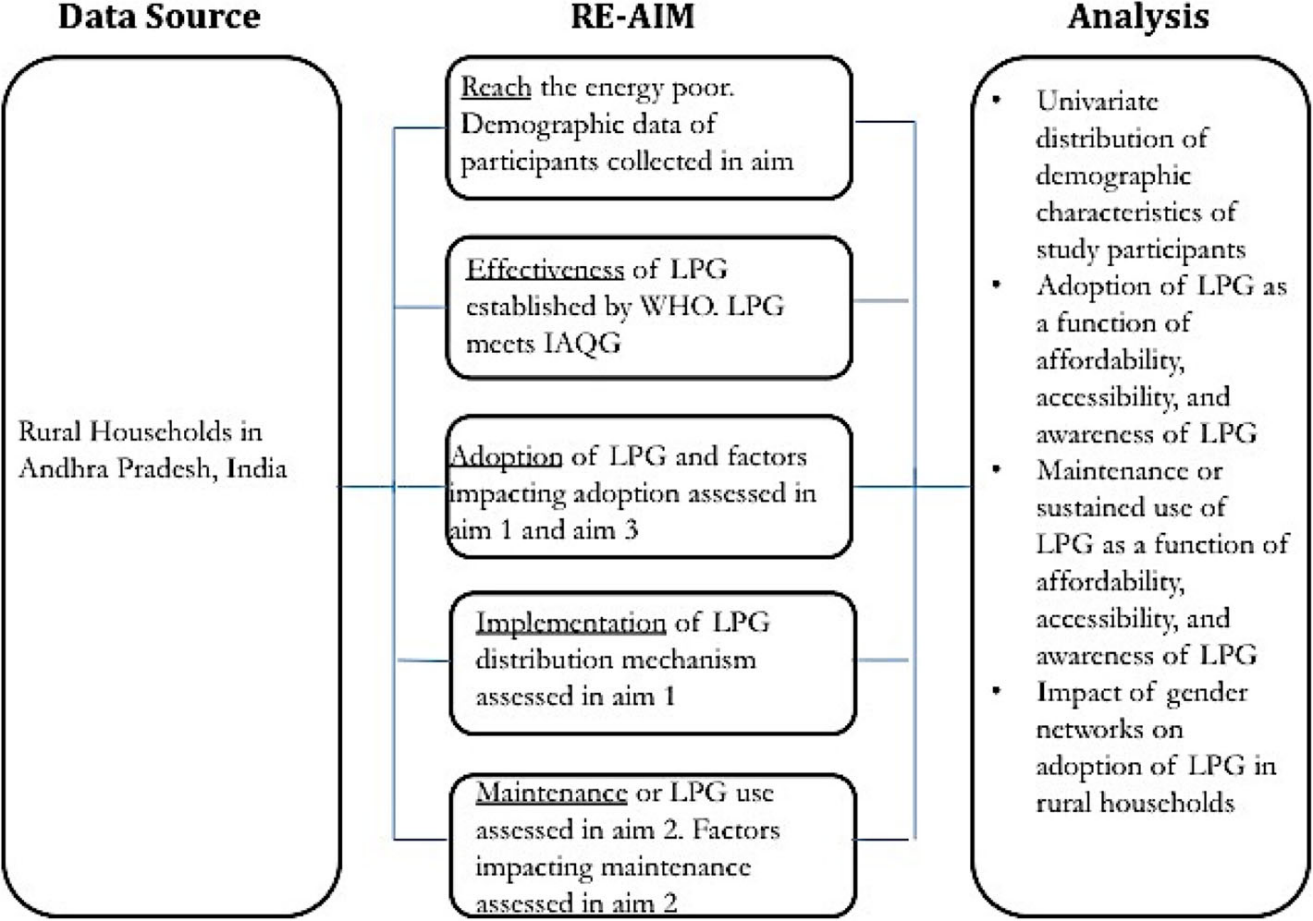
RE-AIM framework to analyze determinants of adoption and sustained use of LPG. This study is guided by the RE-AIM (Reach, Effectiveness, Adoption, Implementation, and Maintenance) implementation science framework. This protocol paper highlights the application of the RE-AIM framework in our study of LPG adoption and sustained use in poor households. This figure synthesizes the placement of the three aims and corresponding analyses of the study within the RE-AIM framework

The RE-AIM framework emphasizes multilevel and concurrent examination of household and organizational level factors driving adoption, implementation, and maintenance of health interventions, instead of an isolated examination of such levels [33, 34, 36]. It seeks to address the ‘voltage drop’ as interventions move from efficacy testing to real world sustainment [37]. The barriers and enablers of adoption and use occur at individual and possibly at organizational levels. The study protocol allows us to examine both these levels [34]. Similarly, individual and organizational level factors may influence effectiveness of an intervention [34, 36]. While affordability is a household level factor, accessibility and awareness of LPG cut across household and organizational level factors. RE-AIM framework allows us to examine how affordability, accessibility, and awareness at multiple levels influence adoption and sustained use of LPG [33, 34, 36] in study aim 1 and study aim 2. In addition, gender networks in communities also influence uptake and use of health interventions [38]. Aim 3 explores the relative influence of personal gender networks of males and females in selected households and their impact on LPG adoption. Figure 2 synthesizes the placement of the three aims and analyses of our study within the RE-AIM framework.

The dissemination of LPG cooking systems in these energy poor communities are undertaken as part of pro-poor projects by the state government and national government in India to reduce HAP. Evaluation of these government projects is crucial as the Government of India has plans to scale up and adopt strategies to push LPG to the rural interiors of the country. A successful scale up requires a nuanced understanding of multiple factors impacting the intervention. This study is situated within the domain of implementation science, and will employ the RE-AIM evaluation framework to systematically explore the factors impacting adoption of LPG. The study will examine multiple household, organizations, and network related drivers that could enable LPG adoption. These factors could be tested in multiple geographies to strengthen the evidence base for scaling up LPG dissemination. It is expected that the study will provide evidence that there are multiple and concurrent determinants of LPG adoption. Understanding and addressing these determinants is crucial for fostering adoption of cleaner cooking systems such as LPG in energy poor communities. Using the RE-AIM framework to undertake this study serves two purposes: 1) factors analyzed against each of the RE-AIM dimensions will be available to frame and study adoption and sustained use of cleaner cooking systems in other geographies and contexts; and 2) factors examined in this study could then be tailored and tested in the adoption of other evidence-based interventions to improve the health and wellbeing of poor communities. For instance: sustained use of contraceptives in Uganda, toilets in Bangladesh, mosquito nets in parts of Africa, and mobile technologies in rural India.

### Sampling and recruitment

We will undertake a case control study in the rural habitations of Thambalpalle and Peddamandyam mandals (block) in Chittoor district of Andhra Pradesh state in India [27]. Our implementing partner, Foundation for Ecological Security (FES) has extensive field experience working in this region and we will leverage their expertise and understanding of social, economic, and demographic characteristics of the households in these habitations.

We will select an equal number of case and control households for this proposed study (control to case ratio will be 1:1). We will select a sample size of 255 households each for case (LPG adopter households) and control (LPG non-adopter households). A total sample size of 510 households at a 95% confidence level (alpha = .05) will provide a power of 80% to this study at a control to case ratio of 1. This computation assumed that the populations mean difference in monthly income was 545.35 India National Rupee (INR), and the common within-group standard deviation was 2199.26 INR per month. In the absence of previously published studies on rural income in this region, where the study is to be undertaken, the power computation was conducted using a baseline income data from another recently concluded NIH funded R21 (WUSTL IRB ID# 201207016) randomized controlled trial (RCT) on cookstoves. This study will be conducted in the same region where the RCT was undertaken. To calculate the sample size, we used the Power and Precision 4 software. The criterion for significance (alpha) was set at 0.05. The test was 2-tailed, and so an effect in either direction will be interpreted. With the proposed sample size of 255 each for case and control group, the study will have power of 80% to yield a statistically significant result. It is also assumed that this effect size is reasonable; an effect of this magnitude could be anticipated in this field of research. On average, a study of this design would enable us to report the mean income difference with a precision of 95% confidence level.

We use a multistage random sampling to select households in these rural habitations. To facilitate sample selection, we develop an exhaustive list of habitations (and households in these habitations) with four key variables: 1) distance to nearest LPG distribution center; 2) dominant caste of the habitation; 3) number of households in each habitation; 4) presence of LPG adopters in these habitations. A multistage sample selection process will be conducted using the following steps in their respective order:

1. **Sample of habitations:** We will use stratified random sampling to select the list of villages for the study. The selection criteria are:

- *Proximity to the nearest LPG distribution center:* Based on the distance from the nearest LPG refilling and distribution center, we divide the exhaustive list of habitations (i.e. habitations population list) in two sub-groups: near to the center and far from the center, by taking a median split of the distance of the habitation from the LPG distribution center.
- *Dominant caste of the habitation:* We divide the habitations population list into three sub-groups: General Caste, Other Backward Castes (OBC), and Scheduled Castes/Scheduled Tribes (SC/ST).

These two stratifying variables and their subgroups will lead to six distinct strata (2subgroups*3subgroups). We will divide the habitations population list across these six distinct strata. We will select a total sample of 35 habitations from these six distinct strata. Table 1 shows an indicative list of the six distinct strata that will be used to select the final 35 habitations.

2. We have data on the number of LPG adopters and non-adopters from each of these selected habitations. We will use quota sampling to select households from each of these habitations. We will randomly select 255 LPG adopter households (case) from these 35 habitations. Subsequently, we will select as many non LPG adopter households (control) as we have LPG adopters (case) across each of the habitations. This will ensure a case control design in every habitation, with a control to case ratio as 1, and a total sample size of 510 households.

**Table 1:** Exhibit list of stratified random sample of habitations

Strata	Proximity to LPG distribution centers	Dominant caste
1	Near	General
2	Near	OBC
3	Near	SC/ST
4	Far	General
5	Far	OBC
6	Far	SC/ST

In case we do not get the required sample size of 510 households in these 35 habitations, we will repeat steps 1 and 2 for selecting additional habitations to reach the required sample size.

### Study participants (*N* = 510; 255 from each group)

The inclusion criteria for the study participants are: rural household with an adult male and female member (>18 years age), woman respondent who is able to provide consent for the study, the woman respondent is the primary cook of the house, senior most male respondent (or primary male decision maker in the household) who is able to provide consent for the study, women respondent residing in the household for the last 12 months, women respondent plans to reside in the household for at least 12 months from the date of enrollment for the study. An additional inclusion criterion for case group (LPG adopter households) is: household has received the first LPG cylinder in the last 12 months from the date of enrollment for the study.

### Data collection

#### Aim 1: To understand how rural LPG adopters vary from other rural households on factors of affordability, accessibility, and awareness of LPG

A structured household adoption questionnaire will be used to record data on social, economic, and demographic characteristics. Women (primary cook) from each household will be the respondent for this questionnaire. In addition to household demographic characteristics, the questionnaire will record data pertaining to the 3 significant drivers impacting LPG adoption: affordability, accessibility, and awareness (3As) of LPG. The categorical outcome variable for aim 1 will be adoption of LPG at the time when data collection is undertaken for the household. Candidate variables on affordability include household income, upfront costs to acquire LPG, access to bank loans, facility of payment in installments for LPG purchase and refill, regularity of income (in past 12 months), and women’s financial autonomy. Candidate variables on accessibility include distance of habitations and households from LPG distribution centers, presence of paved roads to LPG distribution centers, and nature of LPG cylinders’ home delivery system. Candidate variables for awareness include government promotion campaigns, local self-government *(Gram Sabha)* awareness drives, and participation of women in self-help groups. In addition, we will capture data on how organizations at the community level, including government entities, and LPG distributors shape household adoption and sustained use of LPG. Candidate organizational factors are irregular supply and bureaucratic hurdles to apply for LPG, which will be assessed from the perspective of households. Key control variables that will be recorded are caste, household size, months of LPG ownership (in case group), educational status of household head, and free availability of biomass. This adoption questionnaire for aim 1 will be administered to all 510 households immediately after their enrollment in the study.

#### Aim 2: To determine how affordability, accessibility, and awareness affect sustained use of LPG in adopter households

To study sustained use, we will focus on group 1 (LPG adopters) of our sample. SUMS will be used to measure duration of use (a measure of maintenance) of clean cooking technologies of the study participants. We will use SUMS for both the LPG stoves and traditional cookstoves in a randomly selected subset of 100 households from group 1 (LPG adopters) [31]. We will receive SUMS data on: 1) duration of use of these stoves; 2) extent of use of LPG stoves relative to traditional stoves. We will monitor the use of these stoves for 12 months. SUMS data will be recorded and downloaded every two weeks from each of the 100 households by a trained field person. We will download data using a probe connected to a laptop computer via USB port. Files will be uploaded to our database management system (RedCap). Informed consent will be obtained from the households to continue using the stoves as they do routinely. SUMS will provide continuous data on stove usage in 100 households. For analysis purposes, we will also dichotomize this data as: 1) exclusive LPG users, and 2) stackers. Households using LPG at least 80% of the cooking duration during the monitoring period of 12 months will be characterized as exclusive LPG users. This metric will facilitate comparison of the results of this study with other LPG adoption studies currently underway in different parts of the world [32]. We will use a SUMS data log sheet to record the biweekly SUMS data on sustained use. In addition, we will undertake a follow up survey in these 100 households. During the monitoring period (post enrollment of households) status of 3As in the habitations might change. This requires a follow up survey at the end of 6 and 12 months of the monitoring period. Follow up survey will have questions pertaining to the 3As, focused on the monitoring period. Follow up questionnaires and SUMS data collection will be administered to 100 LPG adopter households.

#### Aim 3: To evaluate the relative influence of gender networks on LPG adoption in rural households

Personal social networks of individuals in communities influence awareness levels, which in turn drive adoption, implementation, and maintenance of stoves [39]. Implementation of health interventions are impacted by the personal networks of men and women in the households [38, 39]. Both men and women play a critical role in adopting, implementing, and maintaining cleaner cooking technologies like LPG [39]. Thus, it is critical to assess the personal networks of study participants to ascertain how they influence (or get influenced by) decisions related to making a fuel switch. To generate personal network data, we will use a personal network survey to probe the women (primary cook) and the adult male (or primary male decision maker) of 100 households each in the case and control groups. This approach is novel in understanding the role of gender dimensions and the peer effects impacting their fuel choice. Personal social networks, or egocentric networks, focus on the structure and composition of the networks surrounding a target individual [40]. We will use a well-established ego-centric network survey instrument to measure personal networks of men and women. The gendered network data will be collected as follows: 1) the survey will begin with three name generator questions to prompt identification of individuals who give advice, socialize, and support the respondent. 2) After eliciting the network members, a second set of questions will be performed to evaluate the strength of the connections (tie strength) between the respondent and the individuals identified by the respondent; 3) subsequently, the strength of the connections between the network members identified will be probed; 4) finally, characteristics of the network members identified will be solicited (e.g., their demographics, income, household size, and cooking habits).

### Research instruments

A summary of the research instruments is provided in Table 2. Each of the research instruments will be drafted in English and will be translated into Telugu (local language of the region in India).

**Table 2:** Data collection instruments

Conceptual domains	Aims	Research instrument	Method or Measure	Data source	Sample size
Adoption of LPG	Aim 1	Adoption questionnaire	Structured interview	Households: women	LPG adopter households (case): 255; Non-LPG adopter households (control): 255
Sustained use of LPG	Aim 2	SUMS	Stove temperature monitoring	Households: LPG and traditional stoves	LPG adopter households (case): 100
Sustained use of LPG	Aim 2	Follow up questionnaire	Structured interview	Households: women	LPG adopter households (case): 100
Personal social networks of LPG adopters and non-LPG adopters impacting adoption of LPG	Aim 3	Social network survey questionnaires for women and men	Structured interview	Households: women, men	LPG adopter households (case): 100 Non-LPG adopter households (control): 100

### Data analysis

#### Aim 1: To understand how rural LPG adopters vary from other rural households on factors of affordability, accessibility, and awareness of LPG

We will conduct preliminary univariate and bivariate data analyses. The categorical outcome variable for aim 1 (adoption of LPG: yes/no) will be regressed on the predictors (3As) recorded from the adoption questionnaire. We will have two levels of data from the household adoption questionnaire covering information on the 3As: level 1) household level characteristics such as income, women’s financial autonomy, household size, or caste; and level 2) community level characteristics such as LPG cylinders’ home delivery facility, presence of paved roads, government promotion campaigns, or distance of LPG distribution centers from the habitation. Depending on the values of intra class correlation (ICC) coefficient (if higher than the accepted norm of 5%), we will conduct additional analyses. We will use these two levels of data to conduct a multilevel regression modeling (using R version 3.0.3) with habitations as grouping variable, to predict these higher-level influences of 3As on adoption of LPG among rural households. We will also analyze the interaction effect of 3As on LPG adoption.

#### Aim 2: To determine how affordability, accessibility, and awareness (3As) affect sustained use of LPG in adopter households

We will conduct two types of analyses:

1. We will analyze the usage pattern data (using SUMS data log sheet) from LPG stoves and traditional stoves for the monitoring period of 12 months. This trend of usage pattern (without any covariates) will be analyzed for the monitoring period of 12 months using R version 3.0.3.
2. The households will be dichotomized into exclusive LPG users and stackers depending on their usage pattern. Households using LPG at least 80% of the cooking duration during the monitoring period of 12 months will be characterized as exclusive LPG users. The dichotomous outcome will then be regressed on the 3As data from follow up questionnaire data. Preliminary univariate and bivariate data analyses will precede regression analyses. We will use R version 3.0.3 for analyses.

#### Aim 3: To evaluate the relative influence of gender networks on LPG adoption in rural households

We will use personal network data of males and females in both the adopter and non-adopter groups from our sample of households. We will analyze network structure and composition characteristics. For structure, we will explore structural holes (constraint, effective size and efficiency) of social networks across the case and control groups [41]. We will also assess the network density, which describes the number of actual connections compared to the number of potential connections in the network [39, 42]. For composition analysis, we will assess the proportion kin in the networks, and variation across other characteristics (age, cooking habits). We will test the hypothesis that men tend to initiate information and disseminate them to longer distances on issues of cleaner stoves adoption [39]. However, we will also be able to examine if the gender balance shifts and women dominate men in information exchange at closer distances.

### Limitations

There are limitations of this study. Each of these limitations is briefly considered below.

1. **Study design:** The retrospective nature of aim 1 and aim 3 in the study for LPG adopters might lead to concerns of recall bias, which may limit the accuracy of participants’ memories on factors of 3As, which impacts adoption. However, engaging a large sample size of 510 households in such geographically proximal households might reduce the issues of recall bias [43]. Similarly, the retrospective nature of data from follow-up questionnaire for aim 2 might also be subject to recall bias.
2. **Data analyses:** Regression of sustained use (SUMS data) on its determinants (3As) (from follow up questionnaires) will be done for a small sample size of 100 households. Smaller sample size might preclude us from conducting multilevel analyses by controlling for institutional level predictors.
3. **Exploratory study:** There is limited empirical evidence available on the impact of 3As on adoption and sustained use of LPG. This study is primarily exploratory and developmental in nature. However, it is expected that this study will inform future research to develop and test the effectiveness of implementation strategies for LPG adoption and use in resource poor settings within the RE-AIM framework. In addition, findings from the study will provide insights on the estimate of effect size to facilitate a larger R01 study on the impact of 3As on LPG adoption and sustained use in such communities.

## Discussion

In this study we bring three novel approaches together to gain new insights:

1. Apply the RE-AIM (Reach, Effectiveness, Adoption, Implementation, and Maintenance) framework to explore LPG adoption and use. RE-AIM framework will guide this research and development of measures for reach, adoption, implementation, and maintenance in the clean cooking sector.
2. Delineate adoption and sustained use as separate outcomes and analyze them as a function of affordability, accessibility, and awareness (3As) of LPG.
3. Deploy Social Network Analysis (SNA) to understand how gender networks matter in dissemination of awareness and consequently in adoption of LPG.

In combining these approaches, we will be able to: 1) examine the pooled impact of the 3As on LPG adoption and sustained use, absent in the present stock of research; 2) assess the relative influence of personal gender networks of men and women on the adoption and sustained use of LPG, contributing to our understanding of the role of gender networks in the implementation of clean cooking, 3) understand these effects through the RE-AIM framework to apply our insights toward implementation of cleaner fuels and advance implementation science in the clean cooking sector. This will be the first systematic study to use personal social networks striated by gender to analyze LPG adoption in rural India. Lessons drawn from this study are timely, relevant, and of interest to Government of India’s renewed focus on policy design (including provisions in annual budget 2016) and implementation to expand LPG distribution to the poor in rural India [8, 9, 44]. The Government of India (GOI) has committed to redesigning their LPG policy and distribution to penetrate rural communities using a combination of direct cash transfer programs (PAHAL), campaigns encouraging non-poor to give up LPG subsidies (GiveItUp campaign), and smaller LPG cylinders [9]. Systematic research is needed to undertake a concurrent analysis of the 3As and their corresponding impact on both adoption and sustained use of LPG in rural hosueholds. Careful assessment of the elements of 3As could facilitate a model of a succesful pro-poor strategy for LPG uptake and sustained use.

## Footnotes

1 Broadly, there are four caste groupings in India. They are discussed as follows. **General caste:** Also called open category has no reservation in employment in the central or state government systems. It mainly comprises of three classes out of four in the ancient Hindu Varna system, which are the Brahmins, Kshatriyas and Vaishyas. **Scheduled tribes (STs):** These are tribes that have traditionally lived in the forests. They have traditionally been marginalized and not in the mainstream of the society. They are also known as Adivasis, hence called scheduled tribes as they have been added under a “schedule” of the constitution of India. **Scheduled Castes (SCs):** An economically and socially backward community, they have also been traditionally marginalized. **Other Backward castes or OBCs:** They are also form a large group that is heterogeneous and has been considered by the constitution of India as being economically and socially backward. OBCs, SCs, and STs are provided with job reservations in the central and also in state government systems to increase their representation in the mainstream society, and to simultaneously improve their economic and social well-being. OBCs, SCs, and STs are normally and collectively referred to as lower castes.
